# Intracerebral Hemorrhage and Thrombocytopenia After AstraZeneca COVID-19 Vaccine: Clinical and Diagnostic Challenges of Vaccine-Induced Thrombotic Thrombocytopenia

**DOI:** 10.7759/cureus.17637

**Published:** 2021-09-01

**Authors:** Floor N.H. Wilting, Angela M.M. Kotsopoulos, Anouk C.M. Platteel, Jos A.H. van Oers

**Affiliations:** 1 Department of Neurosurgery, Elisabeth-TweeSteden Hospital, Tilburg, NLD; 2 Department of Intensive Care Medicine, Elisabeth-TweeSteden Hospital, Tilburg, NLD; 3 Laboratory of Medical Microbiology and Immunology, Microvida, Elisabeth-TweeSteden Hospital, Tilburg, NLD

**Keywords:** covid-19, sars-cov-2, astrazeneca vaccine, thrombosis, thrombocytopenia, heparin, chadox1 ncov-19, intracerebral hemorrhage

## Abstract

Vaccine-induced immune thrombotic thrombocytopenia (VITT) is a prothrombotic disorder, which has been described as a rare adverse effect of the adenoviral-vectored coronavirus disease 2019 (COVID-19) vaccines. The diagnosis is confirmed by the detection of anti-platelet factor 4 (PF4) antibodies by enzyme-linked immunosorbent assay (ELISA) or functional assay in individuals with the appropriate clinical history. Here, we report a case of a patient who presented with a severe intracerebral hemorrhage and thrombocytopenia 14 days after receiving the first dose of the Oxford-AstraZeneca COVID-19 vaccine, with negative PF4/heparin antibodies tested with ELISA, but positive heparin-induced platelet activation assay (HIPAA).

## Introduction

Recently, several cases of thrombosis with concomitant thrombocytopenia after vaccination with the Oxford-AstraZeneca coronavirus disease 2019 (COVID-19) vaccine were reported [1-6]. This prothrombotic clinical entity resembling heparin-induced thrombocytopenia (HIT), considered as a rare adverse event of the ChAdOx1 nCoV-19 (AZD1222) vaccine, is known as vaccine-induced immune thrombotic thrombocytopenia (VITT). The reported cases show great overlap in serology and functional assays [1-3,7]. However, we hypothesize that VITT is heterogeneous concerning its clinical manifestation as well as the diagnostic workup based on a case of a 27-year-old woman, who in contrast to previously reported cases presented with a severe intracerebral hemorrhage and had negative enzyme-linked immunosorbent assay (ELISA) HIT assay.

## Case presentation

**Figure 1:**
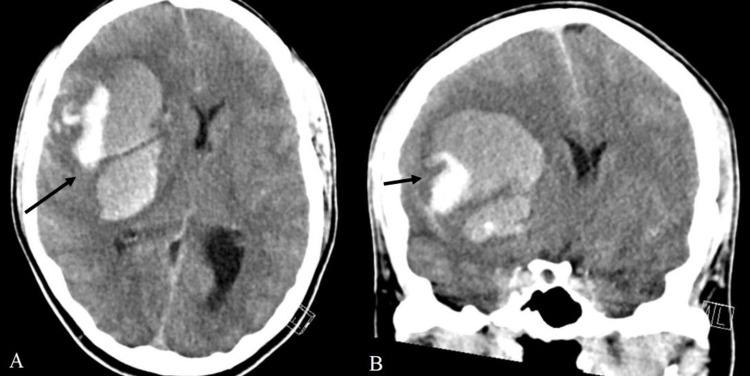
Head CT scan performed on presentation at the emergency department showing a large intraparenchymal hemorrhage in the right hemisphere. (A) Axial plane and (B) coronal plane.

A 27-year-old woman presented at the emergency department with acute left-sided hemiparesis and persistent headache, nausea, and vomiting for six days. The patient had no relevant medical history, in particular, no prothrombotic medical conditions or previous exposure to heparin. Fourteen days before presentation, she received the first dose of the Oxford-AstraZeneca vaccine. Upon arrival at the emergency department, the initial neurological examination showed a Glasgow Coma Scale of E4M6V1 with anisocoria, roving eye movements, left spastic hemiparesis, and bilateral Babinski signs. Cerebral computed tomography (CT) showed a large intraparenchymal hemorrhage in the right hemisphere causing significant mass effect and midline shift, without evidence of an underlying vascular malformation or sinus thrombosis (Figure 1). Laboratory results revealed pronounced thrombocytopenia with extremely elevated D-dimer, elevated international normalized ratio (INR), and low fibrinogen (Table 1).

**Table 1:** Laboratory findings of the patient. ^a^ After administration of 1500IE prothrombin complex concentrate. ^b^ After administration of 3 gram fibrinogen, 1 gram tranexamic acid, two units of platelets, and two units of fresh frozen plasma (FFP). ^c^ After administration of an additional 3 gram fibrinogen, 1 gram tranexamic acid, one unit of platelets, and 1 gram/kg intravenous immunoglobulin (IVIG). aPTT, activated partial thromboplastin time; INR, international normalized ratio.

Parameter (reference value)		At the emergency department ^a^	Intraoperatively ^b^		Ten hours postoperatively ^c^
Platelets	(150–400 10^9^/l)	30	61	87	
D-dimer	(<500 ng/ml)	31,214	-	34,044	
Fibrinogen	(2.00–4.50 g/l)	0.44	1.09	1.15	
Prothrombin time	(10–13.5 sec)	-	16.6	15.6	
aPTT	(24.0–37.0 sec)	34,4	38.2	33.3	
INR	(0.8–1.1)	1.41	1.38	-	

**Figure 2:**
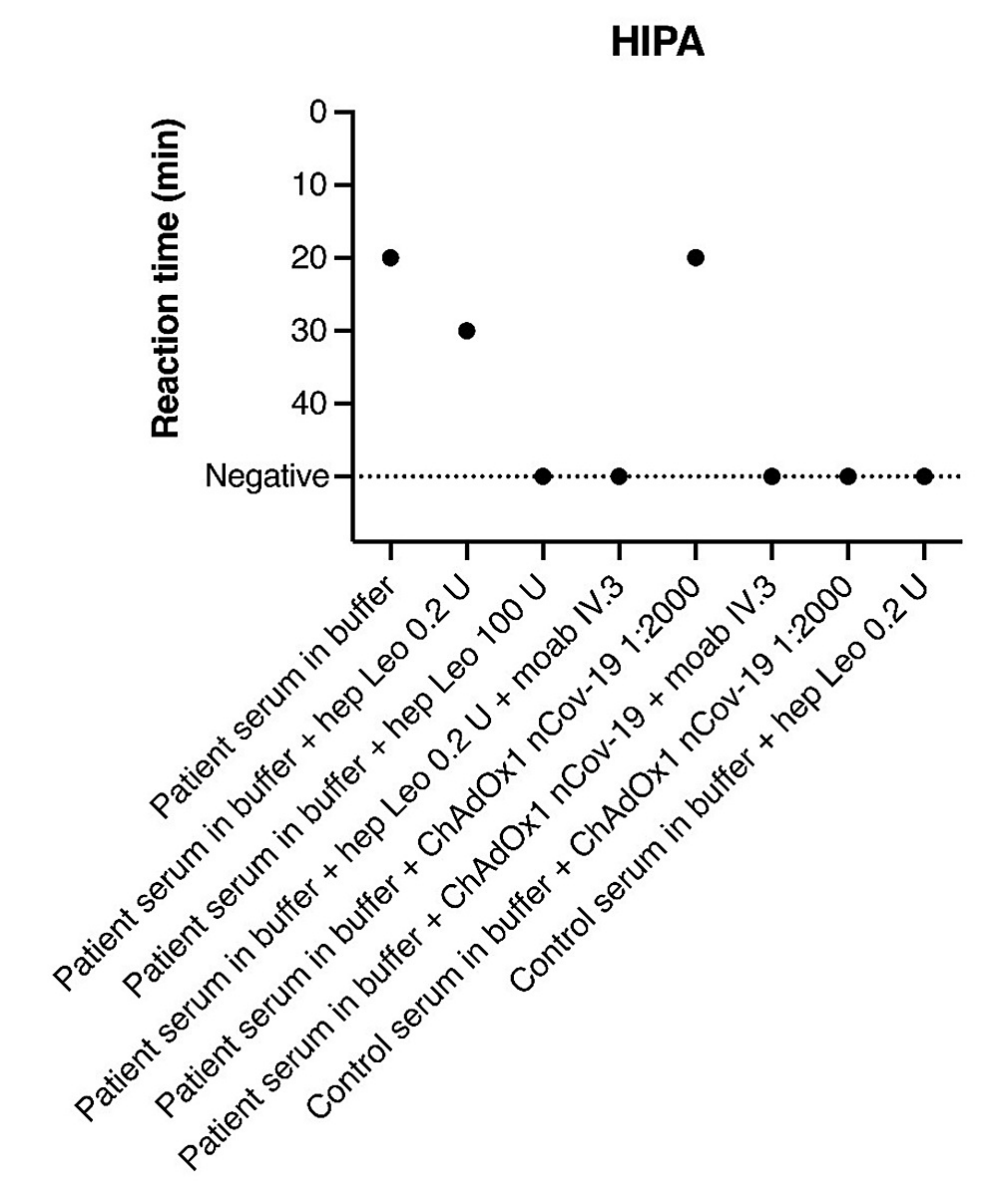
Heparin-induced platelet activation (HIPA) assay reaction times in minutes as single data points. Platelets were co-incubated in buffer with the patient’s serum or control serum. In the different experiments, low (0.2 U) or high (100 U) doses of heparin, the FcγIIa receptor-blocking monoclonal antibody IV.3, or the ChAdOx1 nCov-19 vaccine (diluted 1:2000) were added. Hep Leo = unfractionated heparin by Heparin Leo (Leo Pharma BV, Amsterdam, Netherlands); ChAdOx1 nCov-19 = Oxford-AstraZeneca vaccine; buffer = washing buffer; moab IV.3 = monoclonal antibody IV.3.

Due to rapid neurological deterioration, she underwent emergency craniotomy with evacuation of the intracerebral hemorrhage. She had a pronounced bleeding diathesis, as hemostasis was barely achieved during craniotomy with persistent abnormal coagulation parameters (Table 1), despite administration of both topical (FloSeal, Tisseel, and TachoSil) and systemic agents. In addition, repeat head CT angiography performed on the second day postoperatively revealed concomitant proximal pulmonary embolism. A vaccination-induced HIT-mimicry was suspected. Serology repeatedly showed negative (E < 0.2) immunoglobulin G (IgG) antibodies to platelet factor 4 (PF4)-heparin complexes tested with enzyme-linked immunosorbent assay (ELISA; Asserachrom HPIA-IgG, Diagnostica Stago, Parsippany, New Jersey), but the heparin-induced platelet activation assay (HIPAA; performed as described by Greinacher et al. [1,2]) was positive (Figure 2). This test showed that the patient’s serum activated platelets both in the presence of only buffer as well as the AZD1222 vaccine. The addition of the FcγIIa receptor-blocking monoclonal antibody IV.3 and 100 U heparin blocked the reaction, indicating FcγIIa receptor and PF4 dependence, respectively.

Further testing for thrombotic thrombocytopenic purpura, antiphospholipid syndrome, and microangiopathic hemolytic anemia were negative. Post-operatively, the patient regained consciousness, while the severe hemiparesis persisted. The patient was treated with 1 g/kg intravenous immunoglobulin and fondaparinux (indirect factor Xa inhibitor), followed by argatroban (direct thrombin inhibitor), after which the thrombocytopenia and the coagulation disorders eventually resolved. After seven days of admission to the ICU, she was transferred to the nursing ward, where the argatroban was eventually converted into edoxaban. Finally, after a total hospitalization of 25 days, the patient was discharged to a rehabilitation center for further recovery.

## Discussion

The pathogenic mechanism of COVID-19 vaccine-induced thrombotic thrombocytopenia is recently described in a study by Greinacher et al. [1] as a prothrombotic disorder resembling heparin-induced thrombocytopenia caused by platelet-activating. Nonetheless, the laboratory conditions in patient serum-induced platelet activation differ. Following various published VITT reports, some studies try to describe a potential diagnostic strategy of patients with suspected VITT [2,8]. These strategies recommend testing for PF4/heparin IgG by ELISA in case of thrombosis or thrombocytopenia after recent vaccination with ChAdOx1 nCov-19, followed by a functional assay (HIPAA or serotonin release assay) in case of a positive ELISA. However, this case might not be diagnosed if the diagnostic workup for VITT was strictly followed, due to the negative PF4/heparin antibodies. Whether the latter relies on false-negative ELISA, or a reaction mediated by the presence of PF4-related chemokine antibodies, such as interleukin-8 (IL-8) or neutrophil-activating peptide-2 (NAP-2), is to be discovered [9,10]. Next to the negative ELISA, the atypical clinical course with a severe intraparenchymal hemorrhage as the primary manifestation of VITT can be misleading.

## Conclusions

Our data show a possible VITT with an atypical course and emphasizes the challenges in management and diagnosis. However, as VITT is rare, further research collaboration is needed to elucidate the underlying mechanism.
